# Potential benefits of triage for the trauma patient in a Kenyan emergency department

**DOI:** 10.1186/s12873-018-0200-7

**Published:** 2018-11-29

**Authors:** Maria Lampi, Johan P. E. Junker, John S. Tabu, Peter Berggren, Carl-Oscar Jonson, Andreas Wladis

**Affiliations:** 1 Center for Disaster Medicine and Traumatology, and Department of Clinical and Experimental Medicine, Linköping University, Linköping, Sweden; 2 Department of Disaster Risk Management, Moi University College of Health and Science, Eldoret, Kenya

**Keywords:** Triage, Trauma, Emergency department

## Abstract

**Background:**

Improved trauma management can reduce the time between injury and medical interventions, thus decreasing morbidity and mortality. Triage at the emergency department is essential to ensure prioritization and timely assessment of injured patients. The aim of the present study was to investigate how a lack of formal triage system impacts timely intervention and mortality in a sub-Saharan referral hospital. Further, the study attempts to assess potential benefits of triage towards efficient management of trauma patients in one middle income country.

**Methods:**

A prospective descriptive study was conducted. Adult trauma patients admitted to the emergency department during an 8-month period at Moi Teaching and Referral Hospital in Eldoret, Kenya, were included. Mode of arrival and vital parameters were registered. Variables included in the analysis were Injury Severity Score, time before physician’s assessment, length of hospital stay, and mortality. The patients were retrospectively categorized according to the Rapid Emergency Triage and Treatment System (RETTS) from patient records.

**Results:**

A total of 571 patients were analyzed, with a mean Injury Severity Score of 12.2 (SD 7.7) with a mean length of stay of 11.6 (SD 18.3) days. The mortality rate was 1.8%. The results obtained in this study illustrate that trauma patients admitted to the emergency department at Eldoret are not assessed in a timely fashion, and the time frame recommendations postulated by RETTS are not adhered to. Assessment of patients according to the triage algorithm used revealed a significantly higher average Injury Severity Score in the red category than in the other color categories.

**Conclusion:**

The results from this study clearly illustrate a lack of correct prioritization of patients in relation to the need for timely assessment. This is further demonstrated by the retrospective triage classification of patients, which identified patients with high ISS as in urgent need of care. Since no significant difference in to time to assessment regardless of injury severity was observed, the need for a well-functioning triage system is apparent.

## Background

Beyond communicable and chronic diseases, the mortality rate caused by injuries in Africa is 116 per 100,000 population, compared to 49 per 100,000 population in Europe [1]. In Kenya, which is a middle income country (MIC) in East Africa, the mortality rate caused by injuries is 101 per 100,000 population [1]. It is well known and described that injuries, particularly those sustained through road traffic accidents, are a major cause of death in Africa [1–3]. At least 3000 people are killed annually on Kenyan roads, with an estimated annual road fatality rate of 29.1 per 100,000 population [2]. From a global perspective, injuries in general result in permanent disability among an economically productive young adult population [4]. Although receiving increased attention, implementation of strategies to improve trauma care in low-and middle income countries (LMICs) to address the health burden on the emergency care service is still warranted [5].

To promote trauma care, standardized measurements of trauma severity, outcome and effectiveness of trauma care should be studied [6]. The Injury Severity Score (ISS) has been used for this purpose [7]. Despite LMICs carrying the greatest burden of injury, the implementation of trauma registers and systems for trauma care are lacking [8, 9].

The goal of trauma care is to reduce morbidity and mortality by rapid assessment, stabilization and treatment of the injured. “The golden hour” is a term often used by medical personnel in the trauma care setting to reflect the crucial time period immediately after injury [10]. During this time, rapid treatment in an appropriate facility provides the victim with the greatest chance of survival and minimizes subsequent complications [11]. Waiting time due to overloaded emergency departments (ED) will risk delayed medical intervention [12].

One approach to manage patient flow in the ED setting is the use of a simple triage tool, potentially providing a cost-effective improvement in emergency care [13]. An efficient triage system aims to support medical personnel in identification of life-threatening conditions, performing timely assessments and prioritization according to the severity of the patient’s medical condition [14]. Triage performed in a rapid and accurate fashion is a key element to minimize mortality and morbidity in the ED [15]. A large patient flow, combined with lack of formal triage systems and training results in an increased potential risk for injured patients in EDs [16]. Emergency care, including triage, is a weak part of the health care system especially in LMICs, and improvements there have great life-saving potential [17]. Moreover, previous studies have highlighted that assessing vital signs is essential for identifying the most critically injured patients, and that triage decisions should be based on physiologic parameters [18].

Globally, a wide range of triage systems are used in EDs [19]. Some, but not all, are based on the patients’ vital parameters. One of them is RETTS (Rapid Emergency Triage and Treatment System), a process-oriented triage scale with physiologic parameters such as blood pressure, pulse rate, respiratory rate, blood oxygen saturation, temperature and the reaction level scale [20, 21]. RETTS sorts patients in a color-coded five-level scale, where each level of priority has a defined time limit within which assessment by a physician should begin [22].

Despite the clear need and known benefits of triage, many hospitals in LMICs have forgone triage [13] due to lack of experienced emergency personnel, training and resources [13, 16, 18]. Kenya is no exception. Currently, there is no defined triage system in use at the ED at Moi Teaching and Referral Hospital (MTRH) in Eldoret, Kenya.

The aim of the present study was to investigate how a lack of formal triage system impacts timely intervention and mortality in a sub-Saharan referral hospital. Further, the study attempts to assess potential benefits of triage towards efficient management of trauma patients in one MIC.

## Methods

### Study site

This study was carried out at MTRH ED in Eldoret, Kenya. MTRH, the second national referral and teaching hospital is a tertiary health care facility that attends to all patients including trauma. The hospital is located in western Kenya and has a catchment of over 22 million people from the whole western region of Kenya, parts of eastern Uganda and South Sudan. The hospital can cater to clients in different units such as medical, surgical wards (neuro surgical, orthopedics, general surgery) and pediatrics. The bed capacity at the time of the study was 700, of which six were intensive care unit beds.

### Study design

This was a prospective descriptive study conducted on trauma patients admitted through the ED at MTRH. Adult trauma patients (> 14 years) were included continuously over a period of 8 months from November 2014 to June 2015. A tool to register patient data was designed and administered by research assistants to the nurses manning the ED arrival desk. Variables recorded included patient gender and age, time of arrival, condition at arrival, the mode of transport and type of incident. Descriptive data included the time of the accident, arrival at the ED and interventions performed at the ED. Respiratory rate, pulse, blood pressure, Glasgow Coma Scale (GCS), blood oxygen saturation and body temperature were measured during arrival and admission. The mechanism of injury was recorded on arrival. Also, time spent in the ED before admission and the numbers of treatment days after admission to other departments were registered. A database was designed and included calculation of ISS, [7] waiting time before physician assessment, length of hospital stay, and mortality. As an outcome measure and according to the protocol on discharge, the ISS was recorded 30 days after admission or at the time of discharge. ISS calculations were performed by at least two trauma-experienced physicians. A team of clinicians consisting of a team leader and three other staff (medical officer, nurse, and clinical officer) were responsible for the registration. To validate the database, monthly control audits were carried out for the duration of the data collection period. Each audit included ten randomly selected patient records, which were checked for accuracy in terms of database input and ISS scoring. The audits were performed by two independent specialist physicians and one nurse. Patients were retrospectively categorized according to RETTS [20] using the data logged in the patient records. The RETTS classification was performed according to the raw data extracted from the patient charts. As such no interpretation of the data was performed.

### Procedure

A pilot survey of 29 trauma patients was conducted between October 21 and November 2, 2014, at MTRH private wing to establish routines for data collection and eliminate any potential pitfalls of the main study. The results from the pilot study motivated some changes to the data collection design. Hence, the 29 patients from the pilot study were not included in the main study. Before the main data collection period started, all physicians, nurses and clinical officers at the ED attended four 1-h sessions on the aims of the study and were instructed on how to obtain data, and how to use the data collection tool.

### Inclusion/exclusion criteria

According to MTRH routines, emergency patients are directed to two diverse emergency entrances located in different buildings within the hospital area depending on age. Patients 15 years and above were categorized as adults, seen at the ED and included in the study. Those brought in dead, revisits, or referred patients who had major interventions at referring facilities were excluded. Patients were followed through the ED to the receiving facilities (wards, intensive care unit and theatre) until discharge or evaluation 30 days after admission.

### Data analysis

Statistical analyses were performed using Prism 7.0 (GraphPad, LaJolla, CA). Comparisons between triage categories according to RETTS were performed using a non-parametric Kruskal-Wallis test with a Dunn’s multiple comparisons post test. A *p* value of < 0.05 was considered statistically significant. A non-parametric Spearman test was used to assess correlation between ISS and time to assessment.

### Ethical considerations

The Institutional Research and Ethics Committee (IREC) at MOI University and MOI Teaching and Referral Hospital reviewed and approved the research proposal and has been granted a Formal Approval Number (FAN: IREC 1263). All patient records and data were handled with strict confidentiality by the clinical research team conducting the study as well as the clerk managing the database. Before any data analysis, all patient data were de-identified. The study is part of a project registered at Clinicaltrials.gov (no NCT02303613).

## Results

### Participants

During the 8-month study period, 36,369 patients presented at the ED at MTRH; 700 trauma patients who were admitted were evaluated for inclusion in the study; 129 (18%) patients were excluded according to the exclusion criteria and incomplete registration, resulting in a total of 571 trauma patients included in the analyses.

**Fig. 1:**
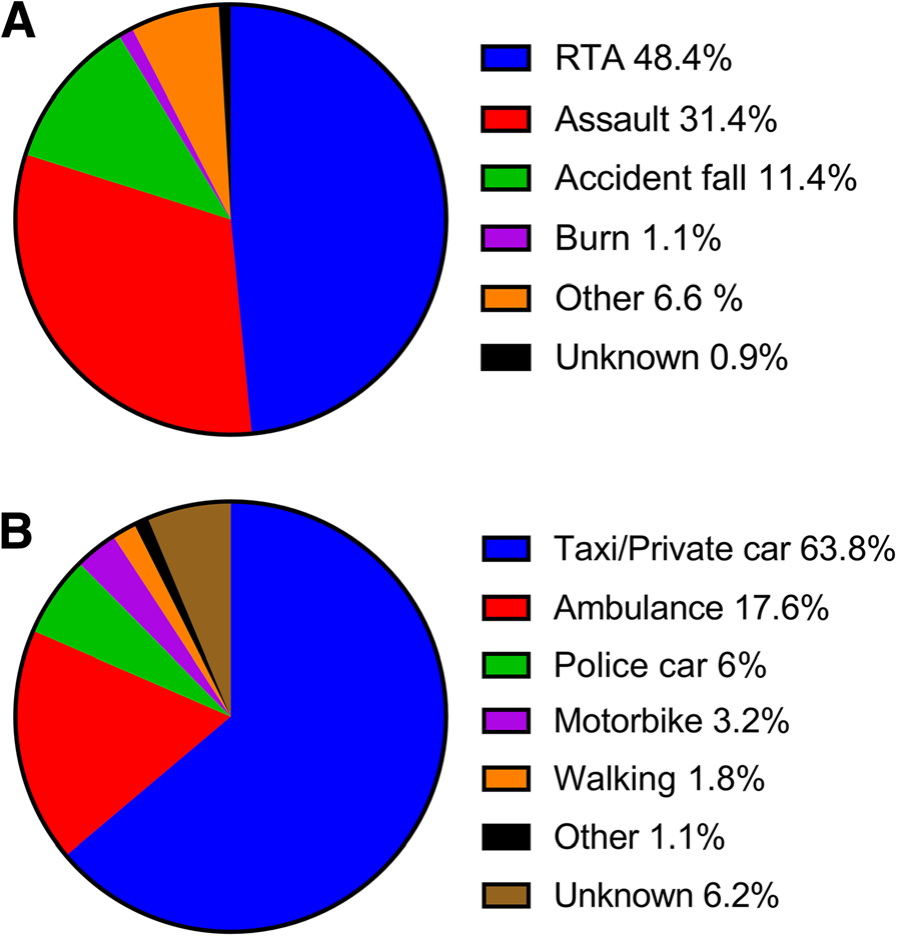
**a** Distribution of patients according to incident type. **b** Illustrating the patients’ arrival mode to the ED

Most patients were male (85%), aged 34 ± 14 years (mean ± standard deviation (SD)). The average length of stay at the hospital was 12 ± 18 days (mean ± SD), (range 0–212 days) with an average ISS of 12 ± 8 (mean ± SD) (Table 1). Among the included 571 trauma patients admitted to the ED, Road Traffic Accidents (RTAs) were the most common mechanism of injury reported, accounting for 48.4% of admissions followed by assault (31.4%) (Fig. 1a). Almost 70% of the patients arrived at the ED by taxi, private car, or police car; 17.6% of the patients were transported in an ambulance (Fig. 1b).

**Table 1:** Characteristics of the 569 trauma patients included in the study

	Number	Percent	Mean ± SD
Sex			
Male	479	85	33.5 ± 13.5 years
Female	87	15	38.9 ± 16.7 years
Age			34.1 ± 14.1 years
Length of stay			11.6 ± 18.3 days
Injury Severity Score			12.2 ± 7.7
Mortality	10	1.8

Retrospectively, all patients were color-coded according to RETTS based on recorded vital parameters (Table 2). A Spearman test assessing the correlation between ISS and time to assessment revealed an r value of − 0.041 with a *p* value of 0.43, yielding no significant correlation.

**Table 2:** Actual time to assessment compared to recommendations defined by RETTS

Color code according to RETTS	Time to assessment (min), mean (95% CI)	Maximum time to assessment as defined by RETTS	Injury severity score, mean ± SD	N (%)
Red	46 (29–62)	Immediate attention	17 ± 11	77 (14)
Orange	92 (55–129)	Within 20 min	11 ± 6	83 (15)
Yellow	79 (52–106)	Within 120 min	12 ± 7	114 (20)
Green	68 (53–82)	Not life threatening	11 ± 7	295 (51)
Total	71 (60–82)		12 ± 8	569

**Fig. 2:**
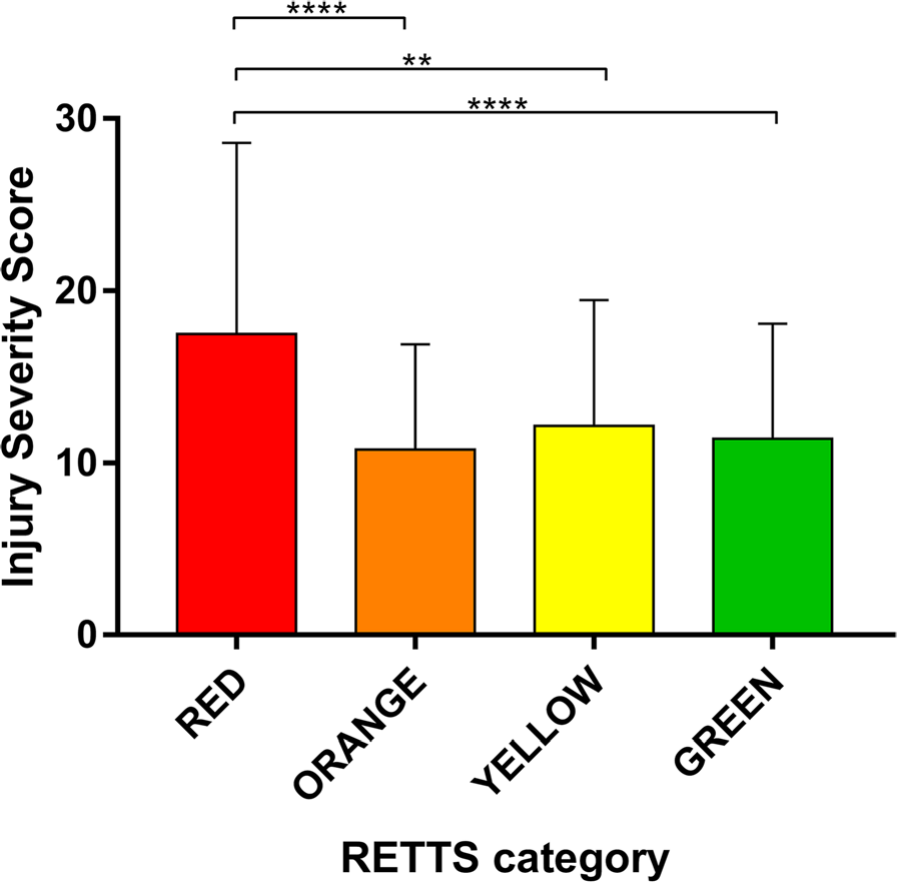
Graph illustrating the Injury Severity Score of patients in relation to RETTS categories. ** denotes *p* < 0.01, **** denotes *p* < 0.001

The retrospective categorization of patients according to the RETTS algorithm was compared with the ISS for each patient using a Kruskal-Wallis test with Dunn’s multiple comparisons post test. This revealed a significantly higher average ISS in the red category than in the other categories (H(df) = 24.47(4), *p* < 0.001) (Fig. 2). No significant differences with regard to ISS were observed when comparing the orange, yellow, or green categories.

The overall mortality rate during the observation period for the patients included in this study was 1.8%; with a total of 10 deaths registered (Table 3). The major causes of death were related to injuries sustained as a result of RTAs. Nine of the deceased patients had severe injuries (ISS > 15).

**Table 3:** Characteristics of the ten deceased patients

Sex	Age (years)	Incident type	Arrival mode	RETTS Color Code	Time to assessment (min)	ISS	Injuries	LOS (days)
Male	31	RTA	Taxi/private car	Green	82	9	Fractured pelvis	2
Male	38	Assault	Police car	Red	19	16	Head and neck injury; GCS 8	23
Male	–	RTA	Taxi/private car	Red	15	25	Head and neck injury; GCS 4	1
Female	–	RTA	Taxi/private car	Red	60	25	Head and neck injury; GCS 3	1
Male	69	Gunshot	Ambulance	Red	30	26	Fractured extremity, head and neck injury; GCS 15	1
Male	43	RTA	Taxi/private car	Yellow	50	41	Fractured extremity, fractured pelvis	5
Female	23	Burn	Ambulance	Green	9	41	Head and neck injury; GCS 15	64
Male	47	RTA	Taxi/private car	Red	0	45	Chest injury, head and neck injury, fractured extremity; GCS 12	2
Male	43	RTA	Taxi/private car	Red	20	50	Injuries to chest and abdomen	1
Female	30	RTA	Taxi/private car	Red	–	57	Head and neck injury, chest injury, fractured extremity; GCS 8	1

*ISS* Injury Severity Score, *LOS* length of stay, *RTA* road traffic accident, *GCS* Glasgow Coma Scale

## Discussion

Triage in the ED aims at ensuring that each patient is treated in order of clinical urgency and that the management is appropriate and timely [14]. Long waiting times can potentially result in harmful delays and thus increase mortality and morbidity [15]. The triage system used retrospectively in the current study has been developed for timely assessment of the injured patient at the ED [22]. Our findings show that trauma patients included in our study and admitted to the ED at MTRH were not assessed in a timely fashion, and that the time frame was outside recommendations postulated by RETTS. This may result in a reduced chance of survival and increased risk of subsequent complications. The time before treatment of the patients included in this study frequently surpasses the golden hour, even without accounting for the time required for transport from the incident to the ED. Although not a statistically significant finding, the severely injured patients (RETTS classification: red, ISS 17) displayed a shorter time to assessment. This may be due to some sort of rudimentary triage used by the clinicians. In the present study, we have just included trauma patients. Other factors that might influence time to assessment must be considered, such as lack of resources and staff, overcrowding of non-traumatic patients and time of the day and year.

Previous studies have described difficulties in implementing complex triage systems such as RETTS in LMIC, one reason being the lack of relevant staff training [23]. This lack of training has been identified as a key factor that negatively influences the accuracy of triage decisions in the ED [24, 25].

A standardized triage system should be implemented to improve trauma care in LMIC. Independent of the specific system implemented, an assessment of its usefulness and validation after implementation is required [26]. Furthermore, implementation of a trauma triage system should be preceded by an evaluation of equipment and resources available to enable medical staff to establish the appropriate triage category for injured patients. Trauma scoring systems such the Kampala Trauma Score (KTS) has been suggested and evaluated as a triage system in a sub-Saharan African trauma cohort, but no system that is validated for clinical decision making currently exist [27].

In the present study, no statistical correlation was found when comparing ISS and time to assessment, which suggests inadequate prioritization of patients. However, the retrospective classification of patients according to RETTS led to identification of the most severely injured patients (ISS > 15) [7], which were categorized as red, thus mandating urgent medical assessment.

Moreover, the mortality rate in the study cohort was only 1.8% (10 patients). These patients had an ISS of 34 ± 15 (mean ± SD). This finding can be compared to a previously published study on trauma in Kenya which reports a mortality rate of 3.5% in patient cohort with a median ISS of 9 [28]. Although the mortality rate described in the present study may seem surprisingly low, the number of patients who died outside the hospital is unknown. Thus, in-hospital deaths in this study most likely account for only parts of the total deaths attributed to traumatic injuries during the study period. Financial constraints and traditional beliefs may prompt relatives to avoid bringing severely injured and dying patients to the hospital. Also, severely injured patients may have been transported to some of the local hospitals surrounding Eldoret.

About 70% of the trauma patients included in the present study arrived at the ED by police car, taxi, or private car, which is in line with findings in previous studies [12, 29]. This mode of arrival shows significant gaps in pre-hospital organization resulting in a delay of definitive care. Traumatic injury is a significant public health problem, with most deaths occurring outside the hospital [30, 31]. Observations from the present study support the notion that improvement in trauma care must include the pre-hospital organization.

## Limitations

The current study was performed at a single ED over a limited time period, and as such the results may not be fully generalizable to other settings. However, several studies performed in LMIC with comparable health care systems and demographics have identified similar challenges and possible improvements [12, 29–35]. Approximately 18% of trauma patients presented at the ED during the study period were excluded from analyses due to inconsistent record keeping and documentation [36].

## Conclusion

The results from this study clearly illustrate a lack of correct prioritization of patients in relation to the need for timely assessment in a sub-Saharan referral hospital. This is further demonstrated by the retrospective triage classification of patients, which identified patients with high ISS as in urgent need of care. Since no significant difference in to time to assessment regardless of injury severity was observed, the need for a well-functioning triage system is apparent.

A high proportion of injured patients arriving to the ED by police, taxi or private car was found in this study, again underlines the need for the establishment of a pre-hospital organization as a part of improving trauma care in Kenya and other LMIC.

To provide adequate trauma care, efforts should be made to continue the ongoing improvement of standardized trauma patient registrations in the pre-hospital and in-hospital setting, not only in Kenya, but also in other LMIC.

## References

[1] World Health Organization (2015). World health statistics 2015.

[2] World Health Organization (2015). Global status report on road safety 2015.

[3] Lozano R, Naghavi M, Foreman K, Lim S, Shibuya K, Aboyans V (2012). Global and regional mortality from 235 causes of death for 20 age groups in 1990 and 2010: a systematic analysis for the global burden of disease study 2010. Lancet.

[4] GBD 2015 Mortality and Causes of Death Collaborators. Global, regional, and national life expectancy, all-cause mortality, and cause-specific mortality for 249 causes of death, 1980–2015: a systematic analysis for the Global Burden of Disease Study 2015. Lancet. 2016:388, 1459–1544. 10.1016/S0140-6736(16)31012-1 PMC5388903 27733281

[5] Yeboah D, Mock C, Karikari P, Agyei-Baffour P, Donkor P, Ebel B (2014). Minimizing preventable trauma deaths in a limited-resource setting: a test-case of a multidisciplinary panel review approach at the Komfo Anokye teaching Hospital in Ghana. World J Surg.

[6] Roy N, Gerdin M, Schneider E, Kizhakke Veetil DK, Khajanchi M, Kumar V (2016). Validation of international trauma scoring systems in urban trauma centres in India. Injury.

[7] Tohira H, Jacobs I, Mountain D, Gibson N, Yeo A (2012). Systematic review of predictive performance of injury severity scoring tools. Scand J Trauma Resusc Emerg Med..

[8] O’Reilly GM (2013). Trauma registries in developing countries: a review of the published experience. Injury.

[9] Wesson HKH, Bachani AM, Wekesa JM, Mburu J, Hyder AA, Stevens KA (2013). Assessing trauma care at the district and provincial hospital levels: a case study of hospitals in Kenya. Injury.

[10] Farrokhnia N, Göransson KE (2011). Swedish emergency department triage and interventions for improved patient flows: a national update. Scand J Trauma Resusc Emerg Med.

[11] Debacker M, Hubloue I, Dhondt E, Rockenschaub G, Rüter A, Codreanu T, et al. Utstein-style template for uniform data reporting of acute medical response in disasters. PLoS Curr. 4:2012, e4f6cf3e8df15a. 10.1371/4f6cf3e8df15a PMC3461975 23066513

[12] Aloyce R, Leshabari S, Brysiewicz P (2014). Assessment of knowledge and skills of triage amongst nurses working in the emergency centres in Dar Es Salaam, Tanzania. Afr J Emerg Med.

[13] Baker T (2009). Critical care in low-income countries. Tropical Med Int Health.

[14] Advanced Trauma Life Support: Student course manual. 9th ed. Chicago, IL: American College of Surgeons; 2012.

[15] Hamilton H, Hodge SD (2011). Trauma, emergency medicine, and the golden hour. Pract Litig.

[16] Chalya PL, Dass RM, McHembe MD, Mbelenge N, Ngayomela IH, Chandika AB (2013). Citywide trauma experience in Mwanza, Tanzania: a need for urgent intervention. J Trauma Manag Outcomes.

[17] Broccoli MC, Calvello EJB, Skog AP, Wachira B, Wallis LA (2015). Perceptions of emergency care in Kenyan communities lacking access to formalised emergency medical systems: a qualitative study. BMJ Open.

[18] Rosedale K, Smith ZA, Davies H, Wood D (2011). The effectiveness of the south African triage score (SATS) in a rural emergency department. South Afr Med J.

[19] Skyttberg N, Vicente J, Chen R, Blomqvist H, Koch S (2016). How to improve vital sign data quality for use in clinical decision support systems? A qualitative study in nine Swedish emergency departments. BMC Med Inform Decis Mak.

[20] Farrohknia N, Castrén M, Ehrenberg A, Lind L, Oredsson S, Jonsson H (2011). Emergency department triage scales and their components: a systematic review of the scientific evidence. Scand J Trauma Resusc Emerg Med..

[21] Henning B, Lydersen S, Døllner H (2016). A reliability study of the rapid emergency triage and treatment system for children. Scand J Trauma Resusc Emerg Med.

[22] Widgren BR, Jourak M (2011). Medical emergency triage and treatment system (METTS): a new protocol in primary triage and secondary priority decision in emergency medicine. J Emerg Med.

[23] Gottschalk SB, Wood D, DeVries S, Wallis LA, Bruijns S (2006). The cape triage score: a new triage system South Africa. Proposal from the cape triage group. Emerg Med J.

[24] Chung JYM (2005). An exploration of accident and emergency nurse experiences of triage decision making in Hong Kong. Accid Emerg Nurs.

[25] Considine J, Botti M, Thomas S (2007). Do knowledge and experience have specific roles in triage decision-making?. Acad Emerg Med.

[26] Christ M, Grossmann F, Winter D, Bingisser R, Platz E (2010). Modern triage in the emergency department. Dtsch Arztebl Int.

[27] Haac B, Varela C, Geyer A, Cairns B, Charles A (2015). The utility of the Kampala trauma score as a triage tool in a sub-Saharan African trauma cohort. World J Surg.

[28] Otieno T, Woodfield JS, Bird P, Hill AG. Trauma in rural Kenya. Injury. 2004;35:S1228–33. 10.1016/j.injury.2004.03.013 15561111

[29] Wesson HKH, Stevens KA, Bachani AM, Mogere S, Akungah D, Nyamari J (2015). Trauma systems in Kenya: a qualitative analysis at the district level. Qual Health Res.

[30] Enumah S, Scott JW, Maine R, Uwitonze E, D’Arc Nyinawankusi J, Riviello R (2016). Rwanda’s model prehospital emergency care service: a two-year review of patient demographics and injury patterns in Kigali. Prehosp Disaster Med.

[31] Nielsen K, Mock C, Joshipura M, Rubiano AM, Zakariah A, Rivara F (2013). Assessment of the status of prehospital care in 13 low- and middle-income countries. Prehosp Emerg Care.

[32] Sunyoto T, Van den Bergh R, Valles P, Gutierrez R, Ayada L, Zachariah R (2014). Providing emergency care and assessing a patient triage system in a referral hospital in Somaliland: a cross-sectional study. BMC Health Serv Res.

[33] Saidi HS, Macharia WM, Ating’a JEO (2005). Outcome for hospitalized road trauma patients at a tertiary hospital in Kenya. Eur J Trauma.

[34] Mulindwa F, Blitz J (2016). Perceptions of doctors and nurses at a Ugandan hospital regarding the introduction and use of the south African triage scale. Afr J Prim Health Care Fam Med.

[35] Odhiambo FO, Beynon CM, Ogwang S, Hamel MJ, Howland O, Van Eijk AM (2013). Trauma-related mortality among adults in rural western Kenya: Characterising deaths using data from a health and demographic surveillance system. PLoS One.

[36] Hung YW, He H (2017). Exploring injury severity measures and in-hospital mortality: a multi-hospital study in Kenya. Injury.

